# Event-Driven Maximum Correntropy Filter Based on Cauchy Kernel for Spatial Orientation Using Gyros/Star Sensor Integration

**DOI:** 10.3390/s24227164

**Published:** 2024-11-07

**Authors:** Kai Cui, Zhaohui Liu, Junfeng Han, Yuke Ma, Peng Liu, Bingbing Gao

**Affiliations:** 1 University of Chinese Academy of Sciences, Beijing 100049, China; lzh@opt.ac.cn (Z.L.); hanjunfeng@opt.ac.cn (J.H.); liupeng1@opt.ac.cn (P.L.); 2 Xi’an Institute of Optics and Precision Mechanics, Xi’an 710119, China; 3 Key Laboratory of Space Precision Measurement, Chinese Academy of Sciences, Xi’an 710119, China; 4 School of Automation, Northwestern Polytechnical University, Xi’an 710072, China; myk@mail.nwpu.edu.cn; 5 Research & Development Institute, Northwestern Polytechnical University in Shenzhen, Shenzhen 518063, China

**Keywords:** maximum correntropy filter, spatial orientation, gyros/star sensor integration, non-Gaussian noise, Cauchy kernel

## Abstract

Gyros/star sensor integration provides a potential method to obtain high-accuracy spatial orientation for turntable structures. However, it is subjected to the problem of accuracy loss when the measurement noises become non-Gaussian due to the complex spatial environment. This paper presents an event-driven maximum correntropy filter based on Cauchy kernel to handle the above problem. In this method, a direct installation mode of gyros/star sensor integration is established and the associated mathematical model is derived to improve the turntable’s control stability. Based on this, a Cauchy kernel-based maximum correntropy filter is developed to curb the influence of non-Gaussian measurement noise for enhancing the gyros/star sensor integration’s robustness. Subsequently, an event-driven mechanism is constructed based on the filter’s innovation information for further reducing the unnecessary computational cost to optimize the real-time performance. The effectiveness of the proposed method has been validated by simulations of the gyros/star sensor integration for spatial orientation. This shows that the proposed filtering methodology not only has strong robustness to deal with the influence of non-Gaussian measurement noise but can also achieve superior real-time spatial applications with a small computational cost, leading to enhanced performance for the turntable’s spatial orientation using gyros/star sensor integration.

## 1. Introduction

The turntable structure can realize the orientation motion of the payload (camera, radar and so on) independently of the spacecraft platform. It plays a vital role for the space tracking and detection system in the aerospace field [1,2]. A two-dimensional turntable is one of the most widely used structures in the above systems. It consists of two rotating platforms, in which their shafting is perpendicular to each other [1,3]. The highly precise orientation measurement of the two-dimensional turntable is an important prerequisite for achieving high stability and enhancing the stability of the space tracking and detection system.

Spatial orientation accuracy refers to the error between the ideal spatial orientation and the actual spatial orientation associated with the inner frame of the turntable after the rotation and transformation of each axis. Due to the inevitable control error, the installation error and other factors in the practical application of the system, there is a deviation between the actual orientation and the ideal orientation of the turntable, which significantly affects the control stability of the turntable [4,5]. Therefore, highly precise orientation measurements of the turntable are particularly important.

Currently, in spatial orientation applications, the star sensor is a commonly used measurement device that can provide high-precision attitude and orientation information [6,7]. However, the operating frequency of the star sensor is quite low and it is easily limited by the time and environmental conditions [7,8]. In contrast, gyros, as an autonomous inertial measurement method, can continuously output high-frequency angular/attitude information with good real-time performance [9,10]. However, gyros have the weakness of error drifts, leading to poor long-term attitude/orientation accuracy [11]. It can be seen that the above two sensors have a good complementary characteristic in terms of attributes, so the gyros/star sensor integration scheme can use the high-precision attitude or orientation information obtained from the star sensor to compensate for the drift of the gyros, achieving high-precision turntable orientation information [12,13].

When the gyros/star sensor integration is implemented for the two-dimensional turntable’s spatial orientation, there are usually two schemes: one is the strap-down installation mode and the other is the direct installation mode [13,14]. In the former, both the gyros and star sensor are installed on the fixed base of the turntable, while in the latter, the gyros are installed in the inner frame of the two-dimensional turntable and firmly connected with the load camera/radar, and the star sensor is installed on the fixed base of the turntable. It should be noted that in many applications, due to the limited space, the star sensor is hardly installed in the inner frame of the turntable because of its large size. In the strap-down installation mode, the turntable’s control stability is easily affected by the turntable error since the gyros/star sensor integration cannot directly perceive the turntable angle. Thus, the direct installation mode has gained increasing attention by scholars in recent years. The research of this paper is also based on the direct installation mode of gyros/star sensor integration.

Nowadays, the data fusion of gyros/star sensor integration is mostly achieved based on Kalman filtering [15,16]. Under the assumption of linear and Gaussian noise models, Kalman filtering can obtain the optimal estimation of a system state [15,16]. However, due to the influence of the spatial light environment, the measurement of star sensors will be contaminated. This makes the measurement noise of gyros/star sensor integration no longer follow a Gaussian distribution, resulting in non-Gaussian noise. In this situation, the performance of Kalman filtering will be significantly deteriorated [17,18].

Research efforts have been dedicated to dealing with non-Gaussian noise involved in the dynamic system. Farahmand et al. proposed a doubly robust smoothing method to provide double robustness that enhances the accuracy and reliability of estimations, particularly in the presence of bias measurement noise covariance and model misspecification [18]. However, the smoothing method is not suitable for a real-time application. Further, Soken et al. established a robust Kalman filter by using a scalar factor to adjust the measurement noise covariance for curbing the influences of inaccurate measurement noise covariances [19]. However, its improvement is very limited since the scalar factor is chosen empirically. Particle filter (PF) and Gaussian sum filter (GSF) are also two typical forms of non-Gaussian filtering [20,21]. Kim et al. developed PF to further extend the non-Gaussian capabilities of filtering by employing sampling and resampling techniques to approximate posterior distributions [20]. Sorenson et al. constructed a GSF by using random sampling to obtain the global approximation of the system state’s posterior probability density, which is not limited to Gaussian noise [21]. However, global approximation requires a large amount of computation, making the above filters difficult to apply for the spatial orientation [22].

As an emerging filtering theory, information theoretic learning has garnered attention for its optimization criteria, which rely on information theoretic measures such as maximum correntropy criterion (MCC) derived directly from data, instead of conventional second-order statistical metrics [23,24]. It exhibits an excellent robustness to handle non-Gaussian noise since it uses the signals’ high-order moments rather than the second-order moments in the minimum mean square error principle. Chen et al. [23] first established the maximum correntropy Kalman filter by using the maximum correntropy criterion. It has superior performance to resist the influences of outliers and non-Gaussian noise for a linear system. Compared to PF and GSF, the filter based on the MCC shows a quite low computational burden. Further, Mohammadi et al. [24] presented a maximum–minimum correntropy criterion for robust and stable gene selection. Gao et al. [25] developed the theory of MCC to handle outliers and non-Gaussian noise to improve the performance of spectral redshift estimation for spectral redshift navigation. In the traditional maximum correntropy (MC) filter, the Gaussian kernel is always used to define the distance between distinct vectors. Nevertheless, it may not always be an optimal selection for the kernel function. An issue arises when the measurement is affected by multi-dimensional non-Gaussian noise, such as heavy-tailed noise in real-world applications. In such cases, the aforementioned MC filters may fail due to the occurrence of singular matrices, causing certain characteristics of the noise to be overlooked and resulting in reduced estimation accuracy [26,27].

To address the drawbacks of Gaussian kernel-based MC filters, a novel Cauchy kernel-based MCC filter is proposed [27,28]. It uses the Cauchy kernel instead of the original Gaussian kernel to describe the distance between different vectors. The Cauchy kernel originated from the Cauchy distribution. Its function curve has a long tail, indicating that the kernel function is very broad and can be used for data with high dimensions. Because of the above reason, the Cauchy kernel-based MC filter shows a superior performance when handling multi-dimensional non-Gaussian noise. It also behaves in a consistently stable manner when a different Cauchy kernel bandwidth is selected [28,29]. Unfortunately, the existing MC filters or Cauchy kernel-based MC filters mostly utilize the fixed-point iteration method to update the estimates of the state, which will burden the computational efficiency [27,30]. For the application of spatial orientation, the real-time performance is a very important property index. Thus, if the MCC is used for the whole filtering process of gyros/star sensor integration, it will increase the unnecessary computational burden, leading to a deteriorated real-time performance. This is because in the time point without measurement fault, i.e., non-Gaussian measurement noise does not exist, the Kalman filter can obtain the optimal estimation result with a lower computational cost than MC filters, while MC filters need to use the fixed-point iteration to update the estimates of the state, requiring a large computational cost.

To address the above problem for a spatial orientation application, this paper presents a novel method of a Cauchy kernel-based maximum correntropy filter that is event-driven to curb the influence of non-Gaussian measurement noise on the solution of gyros/star sensor integration. It first establishes a direct installation mode of gyros/star sensor integration to achieve a high turntable control stability and then derives its associated mathematical model. Further, an event-driven maximum correntropy filter based on the Cauchy kernel is proposed to handle the non-Gaussian noise involved in the measurement of gyros/star sensor integration caused by the complex spatial environment. It also constructs an event-driven mechanism according to the innovation information to reduce the redundant computational cost for the optimization of the filter’s real-time performance. The proposed methodology can achieve robustness to curb the influence of non-Gaussian measurement noise with a superior computational cost compared to the existing MC filters. Simulations and a comparison analysis based on the gyros/star sensor integration for a two-dimensional turntable’s spatial orientation have been carried out to comprehensively evaluate the effectiveness of the proposed filtering method.

## 2. Model of Gyros/Star Sensor Integration for Spatial Orientation

**Figure 1:**
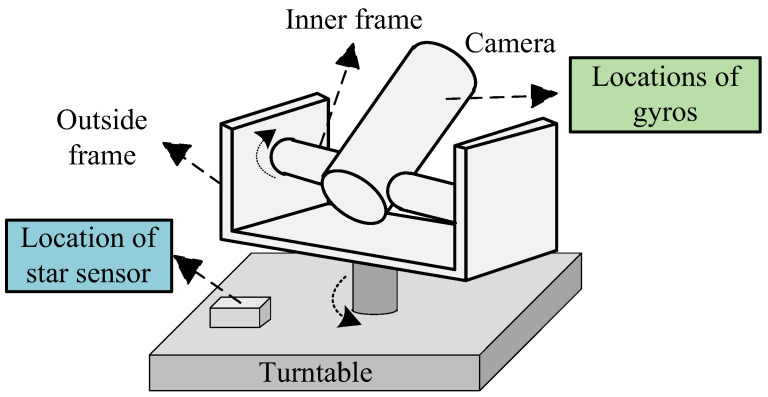
The direct installation mode of the gyros/star sensor integration.

The direct installation mode is used for the integration of the gyros and star sensor, which is described in Figure 1. It can be seen from Figure 1 that the gyros are installed in the inner frame of the two-dimensional turntable and firmly connected with the load camera/radar, and the star sensor is installed on the fixed base of the turntable.

**Figure 2:**
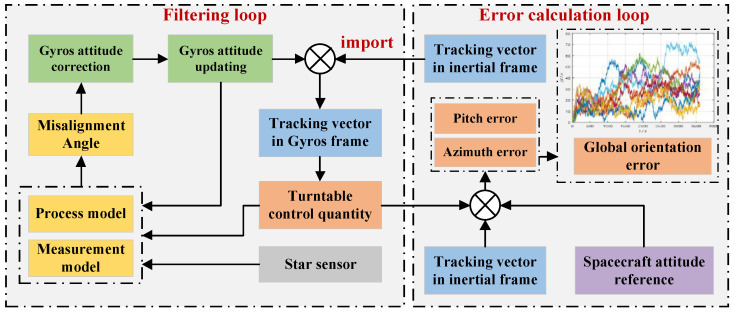
Framework of gyros/star sensor integration for spatial orientation. The different colored lines in the global orientation error plot represent the orientation error curves of multiple repeat tests.

Based on the direct installation mode, the framework of the gyros/star sensor integration used for the spatial orientation is illustrated in Figure 2, which has two loops: a filtering loop and error calculation loop. In the filtering loop, the gyros’ measurement information, the turntable control quantities, and the attitude of the star sensor serve as the input of filter for the error state estimation of gyros/star sensor integration. Then, the estimated gyros’ attitude error is fed back to correct the gyros and the gyros’ attitude is updated for the next time step. In the error calculation loop, the orientation error between the tracking vector (the normalized vector between the spacecraft and target) and the attitude reference of the spacecraft is calculated by using the turntable’s control quantities and the spacecraft’s attitude at each time step to reflect the tracking accuracy.

It should be noted that as shown in Figure 1 and Figure 2, the star sensor realizes the three-dimensional attitude information of turntable base. Since the rotation angles of the turntable’s inner frame and outside frame are known through the pitch and azimuth code discs, the three-dimensional attitude information of the turntable base can be transformed into the turntable’s inner frame (*E*-frame) and inertial frame (*I*-frame). Based on this attitude information, the measurements and measurement model for the gyros/star sensor integration are established. Further, combining the process model with the measurement model, gyros’ platform misalignment angle can be estimated through the filtering process. This estimation will be fed back to the gyros to correct the attitude error. Thus, the star sensor can restrain and correct the gyros’ drift and further enhance the turntable’s spatial orientation accuracy.

Based on the above, the model of gyros/star sensor integration can be further derived and established, which includes the process and measurement models.

### 2.1. Process Model

Denote the inertial frame as the *I*-frame, body frame as the *B*-frame, turntable’s base frame as the *G*-frame, turntable’s outside frame as the *A*-frame, turntable’s inner frame as the *E*-frame and gyros’ composite frame as the *P*-frame.

The *I*-frame is chosen as the navigation frame; thus, the angular relation between the *I*-frame and *P*-frame can be expressed by the attitude matrix [31,32]

(1) $${\dot{C}}_{P}^{I}=C_{P}^{I}\left(\omega_{IP}^{P}\times \right)$$

where $C_{P}^{I}$ is the attitude matrix from the *P*-frame and *I*-frame, ${\dot{C}}_{P}^{I}$ is the differential form of $C_{P}^{I}$, $\omega_{IP}^{P}$ represents the gyros’ output, and $\left(\omega_{IP}^{P}\times \right)$ stands for the skew symmetric matrix of $\omega_{IP}^{P}$, which can be calculated as [33]

(2) $$\left(\omega_{IP}^{P}\times \right)=\left[\begin{array}{ccc} 0 & -\omega_{IPz}^{P} & \omega_{IPy}^{P}\\ \omega_{IPz}^{P} & 0 & -\omega_{IPx}^{P}\\ -\omega_{IPy}^{P} & \omega_{IPx}^{P} & 0 \end{array}\right]$$

where $\omega_{IPi}^{P}$ (i=x,y,z) represents the components of $\omega_{IP}^{P}$ in the directions of *x*, *y* and *z.*

Considering the measurement error, the above attitude matrix should be written as $C_{P}^{I^{\prime }}$, and the $I^{\prime }$-frame and *I*-frame have a platform misalignment angle. Thus, we have the following relationship [32,34]

(3) $$C_{P}^{I^{\prime }}=C_{I}^{I^{\prime }}C_{P}^{I}=\left(I-\phi^{I}\times \right)C_{P}^{I}$$

where $\phi^{I}$ stands for the platform misalignment angle.

Further, according to the definition of platform misalignment angle $\phi^{I}$ in [35], it has a differential relation with the gyros’ zero-bias error, which can be described as follows. The specifics can be seen in [35].

(4) $${\dot{\phi }}^{I}=-C_{P}^{I}\varepsilon^{P}$$

where $\varepsilon^{P}$ is the gyros’ zero-bias error.

It can be seen that the attitude update of the gyros diverges with time due to the existence of gyros’ zero-bias error; thus, we can use the star sensor’s output to estimate and correct the gyros’ attitude errors.

Denote the system state of gyros/star sensor integration as

(5) $$X={\left[\begin{array}{cc} {\left(\phi^{I}\right)}^{T} & {\left(\varepsilon^{P}\right)}^{T} \end{array}\right]}^{T}$$

Based on (5), the process model of gyros/star sensor integration can be described as

(6) $$\dot{X}=\left[\begin{array}{cc} 0_{3\times 3} & -C_{P}^{I}\\ 0_{3\times 3} & 0_{3\times 3} \end{array}\right]X+\left[\begin{array}{c} w_{P}\\ 0_{3\times 1} \end{array}\right]$$

Discretizing (6), we can obtain

(7) $$X_{k}=\left[\begin{array}{c} \phi_{k}^{I}\\ \varepsilon_{k}^{P} \end{array}\right]=\left[\begin{array}{cc} I_{3\times 3} & -C_{P}^{I}\\ 0_{3\times 3} & I_{3\times 3} \end{array}\right]\left[\begin{array}{c} \phi_{k-1}^{I}\\ \varepsilon_{k-1}^{P} \end{array}\right]+\left[\begin{array}{c} I_{3\times 3}\\ 0_{3\times 3} \end{array}\right]W_{k-1}$$

### 2.2. Measurement Model

In the direct installation mode, there is a turntable angle between the gyros and the star sensor. Thus, according to Figure 1, the error matrix can be constructed based on the output of the star sensor

(8) $$M_{Zb}={\tilde{C}}_{I}^{P_{i}}{\tilde{C}}_{B}^{I}C_{G}^{B}C_{A}^{G}C_{E}^{A}={\tilde{C}}_{E}^{P_{i}}$$

where C is the rotation matrix from one frame to another frame.

In the real application, since the installation angle can be calibrated to compensate for its error, the *E*-frame can be seen coincident with the *P*-frame. Further, by using the Rodrigues formula [34], (8) can be further written as

(9) $$\begin{array}{l} M_{Zb}={\tilde{C}}_{E}^{P_{i}}={\tilde{C}}_{P}^{P_{i}}=({\tilde{C}}_{P_{i}}^{P}{)}^{T}\\ \;\;\;\;={\left(I+\frac{\sin\phi^{P}}{\phi^{P}}(\phi^{P}\times )+\frac{1-\cos\phi^{P}}{{\left(\phi^{P}\right)}^{2}}{\left(\phi^{P}\right)}^{2}\right)}^{T}\approx {\left(I+(\phi^{P}\times )\right)}^{T}=\left(I-(\phi^{P}\times )\right) \end{array}$$

where $\phi^{P}={\left[\begin{array}{ccc} \phi_{x}^{P} & \phi_{y}^{P} & \phi_{z}^{P} \end{array}\right]}^{T}$ is the platform misalignment angle between the *P*-frame and *P_i_*-frame computed by the output of star sensor, which can be seen as a small amount; $\phi^{P}=\left|\phi^{P}\right|$ represents its modulus.

According to the definition of the skew symmetric matrix [33], we have

(10) $$\left(\phi^{P}\times \right)=\left[\begin{array}{ccc} 0 & -\phi_{z}^{P} & \phi_{y}^{P}\\ \phi_{z}^{P} & 0 & -\phi_{x}^{P}\\ -\phi_{y}^{P} & \phi_{x}^{P} & 0 \end{array}\right]$$

Thus, expanding (9) yields

(11) $$M_{Zb}=I-\left[\begin{array}{ccc} 0 & -\phi_{z}^{P} & \phi_{y}^{P}\\ \phi_{z}^{P} & 0 & -\phi_{x}^{P}\\ -\phi_{y}^{P} & \phi_{x}^{P} & 0 \end{array}\right]=\left[\begin{array}{ccc} 1 & \phi_{z}^{P} & -\phi_{y}^{P}\\ -\phi_{z}^{P} & 1 & \phi_{x}^{P}\\ \phi_{y}^{P} & -\phi_{x}^{P} & 1 \end{array}\right]$$

Then, the measurement of gyros/star sensor integration can be constructed as

(12) $$Z_{k}=-\frac{1}{2}\left[\begin{array}{c} M_{32}-M_{23}\\ M_{13}-M_{31}\\ M_{21}-M_{12} \end{array}\right]=\left[\begin{array}{c} \phi_{x}^{P}\\ \phi_{y}^{P}\\ \phi_{z}^{P} \end{array}\right]=\phi^{P}$$

where $M_{ij}$ represents the *i*th row and *j*th column element of the matrix $M_{Zb}$.

Using the relationship $\phi^{P}=C_{I}^{P}\phi^{I}$, the measurement model can be established by

(13) $$Z_{k}=\frac{1}{2}\left[\begin{array}{c} M_{32}-M_{23}\\ M_{13}-M_{31}\\ M_{21}-M_{12} \end{array}\right]=\left[\begin{array}{cc} C_{I}^{P} & 0_{3\times 3} \end{array}\right]X_{k}+V_{k}$$

where $V_{k}$ is the measurement noise.

Thus, the model of gyros/star sensor integration for spatial orientation, including the process and measurement models, is achieved.

## 3. Event-Driven Maximum Correntropy Filter Based on Cauchy Kernel

As discussed previously, the star sensor’s measurement is subject to the spatial environment’s interference due to low light intensity. This will make the measurement noise of gyros/star sensor integration in (12) no longer adhere to a Gaussian distribution. In this situation, the traditional Kalman filtering has no ability to deal with the non-Gaussian measurement noise, leading to divergent filtering results. This may cause ineffective correction for the gyros by using the star sensor. Thus, this section establishes an event-driven maximum correntropy filter based on the Cauchy kernel to address the above problem.

### 3.1. Maximum Correntropy Filter Based on Cauchy Kernel

#### 3.1.1. Maximum Correntropy Criterion

In information theory, correlation entropy is often defined as a statistic that describes the similarity between two random variables. Cross-correlation entropy (correntropy) is an extension of the correlation between random variables. It is not only used to measure the second-order information of the joint probability density function but can also measure higher-order moments.

Firstly, the correntropy between the random variables *X* and *Y* is defined as [23]

(14) $$C(X,Y)=E\left[G_{\sigma }(X,Y)\right]=\int G_{\sigma }(X,Y)dF_{XY}(x,y)$$

where $E\left[\;\cdot \;\right]$ represents the calculation of expectation, $G_{\sigma }(\cdot )$ is a shift-invariant Mercer kernel, $F_{XY}(x,y)$ stands for the joint probability distribution of variables X and Y, and ∫⋅ represents the integral operation.

The Gaussian kernel is the most widely used kernel function in Mercer theory, and it is described as [29]

(15) $$G_{\sigma }(X,Y)=G_{\sigma }(X-Y)=\exp\left(-\frac{{\left\|X-Y\right\|}^{2}}{2\sigma^{2}}\right)$$

where σ>0 represents the bandwidth of the Gaussian kernel.

However, the Gaussian kernel may not always be an optimal selection due to the appearance of singular matrices, making the filter break down. In this paper, we use the Cauchy kernel instead of the Gaussian kernel as the kernel function for the correntropy to better capture the heavy-tailed features in the noise. The Cauchy kernel has merits such as being less sensitive to kernel bandwidth and having better stability compared to the Gaussian kernel. It is defined as [27]

(16) $$G_{\sigma }(X,Y)=G_{\sigma }(X-Y)=\frac{1}{1+{\left\|X-Y\right\|}^{2}/\sigma }$$

where σ>0 is the bandwidth of the Cauchy kernel, which is used to adjust the degree of influence of the kernel function on non-Gaussian noise.

In real applications, the true joint distribution function of random variables is usually inaccessible; thus, the sample mean estimation of correntropy is often calculated to approximate the true joint distribution function [28]

(17) $$\hat{C}(X,Y)=\frac{1}{N}\sum_{i=1}^{N}G_{\sigma }(x_{i},y_{i})$$

where N is the sample number, and $x_{i}$ and $y_{i}$ are the sample data of the random variables X and Y.

Correntropy can capture the higher-order moment information of the observed signal, which can be described by the Taylor series expansion of the kernel function [28]

(18) $$\hat{C}(X,Y)=\sum_{n=0}^{\infty }\frac{{\left(-1\right)}^{n}}{\sigma^{n}}E\left[{\left(X-Y\right)}^{2n}\right]$$

It can be seen from (15) and (16) that when X = Y, the correntropy of two random variables reaches its maximum, indicating the strongest similarity between them, which is the core principle of the maximum correntropy criterion. In addition, it can be seen from (18) that the correntropy is a weighted sum of the even-order moments of X − Y. Unlike the minimum mean square error criterion in Kalman filtering, only the second-order moment is considered. Thus, the maximum correntropy criterion can be used to handle the influence of non-Gaussian noises.

Further, from (15) and (16), we can see that the Gaussian kernel gets exponentially closer to 0 as X − Y increases, which leads to an increase in the possibility of matrix singularity. As a comparison, the Cauchy kernel approaches zero much more slowly. Thus, it can effectively reduce the probability of matrix singularity. This is the reason why the Cauchy kernel instead of the Gaussian kernel is used as the kernel function for the correntropy in this paper.

#### 3.1.2. Process of the Maximum Correntropy Filter Algorithm Based on Cauchy Kernel

According to the above-described maximum entropy criterion based on the Cauchy kernel in Section 3.1.1, we can further combine it with Kalman filtering to establish the maximum correntropy filter based on the Cauchy kernel.

As shown in (7) and (13), the system process and measurement models of gyros/star sensor integration can be summarized as the following form

(19) $$\left\{\begin{array}{l} X_{k}=F_{k/k-1}X_{k-1}+W_{k}\\ Z_{k}=H_{k}X_{k}+V_{k} \end{array}\right.$$

where

(20) $$F_{k/k-1}=\left[\begin{array}{cc} I_{3\times 3} & -C_{P}^{I}\\ 0_{3\times 3} & I_{3\times 3} \end{array}\right]$$

The calculation procedure of traditional Kalman filter can be described as

Step 1: Initialization parameter.

(21) $$\left\{\begin{array}{l} {\hat{X}}_{0}=E[X_{0}]\\ P_{0}=E[(X_{0}-{\hat{X}}_{0})(X_{0}-{\hat{X}}_{0}{)}^{T}] \end{array}\right.$$

Step 2: Time update. Using the previous state estimate and its error covariance matrix in time *k* − 1, the state prediction and its error covariance matrix are calculated as [17]

(22) $${\hat{X}}_{k/k-1}=F_{k/k-1}{\hat{X}}_{k-1}$$

(23) $$P_{k/k-1}=F_{k/k-1}P_{k-1}F_{k/k-1}^{T}+Q_{k-1}$$

where $F_{k/k-1}$ is the state transition matrix described by (20); ${\hat{X}}_{k/k-1}$ and $P_{k/k-1}$ are the state prediction and its error covariance at time *k*; ${\hat{X}}_{k-1}$ and $P_{k-1}$ are the state estimation and its error covariance at the previous time *k* − 1; and $Q_{k-1}$ is the process noise covariance.

Step 3: Measurement update. The processes for measurement updates are as follows [17]

(24) $$K^{k}=P_{k/k-1}H_{k}^{T}{\left(H_{k}P_{k/k-1}H_{k}^{T}+R_{k}\right)}^{-1}$$

(25) $${\hat{X}}_{k}={\hat{X}}_{k/k-1}+K_{k}\left[Z_{k}-H_{k}{\hat{X}}_{k/k-1}\right]$$

(26) $$P_{k}=(I-K_{k}H_{k})P_{k/k-1}$$

where $R_{k}$ is the measurement noise covariance; $K_{k}$ is the filtering gain; and $H_{k}$ is the measurement matrix.

However, due to the weak light environment in space, the star sensor’s measurement information is subject to be disturbed, leading to the occurrence of non-Gaussian measurement noise. This further reduces the estimation accuracy of the Kalman filtering and may even cause divergence, resulting in the loss of its ability to correct the gyros.

According to the maximum correntropy criterion based on the Cauchy kernel, we construct a new filtering method to modify the measurement update process of Kalman filtering to handle the influence of non-Gaussian measurement noise. Firstly, based on the system model shown in (19), we have

(27) $$\left[\begin{array}{c} {\hat{X}}_{k/k-1}\\ Z_{k} \end{array}\right]=\left[\begin{array}{c} I\\ H_{k} \end{array}\right]X_{k}+A_{k}$$

where

(28) $$A_{k}=\left[\begin{array}{c} {\hat{X}}_{k/k-1}-X_{k}\\ V_{k} \end{array}\right],\;\mathrm{and}\;E\left[A_{k}A_{k}^{T}\right]=\left[\begin{array}{cc} P_{k/k-1} & 0\\ 0 & R_{k} \end{array}\right]=B_{k}B_{k}^{T}$$

where $B_{k}$ is the Cholesky decomposition of $E\left[A_{k}A_{k}^{T}\right]$.

Multiplying both sides of (27) by $B_{k}^{-1}$ yields

(29) $$Y_{k}=M_{k}X_{k}+E_{k}$$

where $Y_{k}=B_{k}^{-1}\left[\begin{array}{c} {\hat{X}}_{k/k-1}\\ Z_{k} \end{array}\right]$, $M_{k}=B_{k}^{-1}\left[\begin{array}{c} I\\ H_{k} \end{array}\right]$, and $E_{k}=B_{k}^{-1}A_{k}$.

Then, a correntropy cost function can be established as

(30) $$J=\frac{1}{L}\sum_{i=1}^{L}G_{\sigma }\left(E_{k}^{i}\right)=\frac{1}{L}\sum_{i=1}^{L}G_{\sigma }\left(y_{k}^{i}-m_{k}^{i}X_{k}\right)$$

where *L* = *n* + *m* represents the dimension of $Y_{k}$, and *m* and *n* are the dimensions of $Z_{k}$ and $X_{k}$, respectively; $E_{k}^{i}$ and $y_{k}^{i}$ represent the *i*th elements of $E_{k}$ and $Y_{k}$; and $m_{k}^{i}$ is the *i*th row of $M_{k}$.

The optimal state estimation can be obtained using the following equation.

(31) $$\frac{\partial J}{\partial X_{k}}=\frac{2}{\sigma }\frac{1}{L}\sum_{i=1}^{L}G_{\sigma }{\left(E_{k}^{i}\right)}^{2}m_{k}^{i,T}\left(y_{k}^{i}-m_{k}^{i}X_{k}\right)=0$$

After simplification, it is evident that the following result can be obtained [36]

(32) $$\begin{array}{cc} X_{k} & ={\left(\sum_{i=1}^{L}G_{\sigma }{\left(E_{k}^{i}\right)}^{2}m_{k}^{i,T}m_{k}^{i}\right)}^{-1}\left(\sum_{i=1}^{L}G_{\sigma }{\left(E_{k}^{i}\right)}^{2}m_{k}^{i,T}y_{k}^{i}\right)\\ & ={\left(M_{k}^{T}D_{k}M_{k}\right)}^{-1}\left(M_{k}^{T}D_{k}Y_{k}\right) \end{array}$$

where $D_{k}=\left[\begin{array}{cc} D_{k}^{x} & 0\\ 0 & D_{k}^{z} \end{array}\right]$ with

(33) $$\begin{array}{l} D_{k}^{x}=diag\left(\begin{array}{ccc} G_{\sigma }{\left(E_{k}^{1}\right)}^{2} & ,\ldots , & G_{\sigma }{\left(E_{k}^{n}\right)}^{2} \end{array}\right)\\ \;\;\;=diag\left(\begin{array}{ccc} {\left(\frac{1}{1+{\left({\hat{X}}_{k/k-1}^{1}-m_{k}^{1}{\hat{X}}_{k}\right)}^{2}/\sigma }\right)}^{2} & ,\ldots , & {\left(\frac{1}{1+{\left({\hat{X}}_{k/k-1}^{n}-m_{k}^{n}{\hat{X}}_{k}\right)}^{2}/\sigma }\right)}^{2} \end{array}\right) \end{array}$$

(34) $$\begin{array}{l} D_{k}^{z}=diag\left(\begin{array}{ccc} G_{\sigma }{\left(E_{k}^{n+1}\right)}^{2} & ,\ldots , & G_{\sigma }{\left(E_{k}^{n+m}\right)}^{2} \end{array}\right)\\ \;\;\;=diag\left(\begin{array}{ccc} {\left(\frac{1}{1+{\left(Z_{k}^{1}-m_{k}^{n+1}{\hat{X}}_{k}\right)}^{2}/\sigma }\right)}^{2} & ,\ldots , & {\left(\frac{1}{1+{\left(Z_{k}^{m}-m_{k}^{n+m}{\hat{X}}_{k}\right)}^{2}/\sigma }\right)}^{2} \end{array}\right) \end{array}$$

where ${\hat{X}}_{k/k-1}^{i}(i=1,2,\cdots ,n)$ and $Z_{k}^{i}(i=1,2,\cdots ,m)$ are the *i*th elements of ${\hat{X}}_{k/k-1}$ and $Z_{k}$.

By expanding (32), we can obtain

(35) $$\begin{array}{l} X_{k}\;={\left(M_{k}^{T}D_{k}M_{k}\right)}^{-1}\left(M_{k}^{T}D_{k}Y_{k}\right)\\ \;\;={\left({\left(B_{k}^{P,T}\right)}^{-1}D_{k}^{x}{\left(B_{k}^{P}\right)}^{-1}+H_{k}^{T}{\left(B_{k}^{R,T}\right)}^{-1}D_{k}^{z}{\left(B_{k}^{R}\right)}^{-1}H_{k}\right)}^{-1}\\ \;\;\left({\left(B_{k}^{P,T}\right)}^{-1}D_{k}^{x}{\left(B_{k}^{P}\right)}^{-1}{\hat{X}}_{k/k-1}+H_{k}^{T}{\left(B_{k}^{R,T}\right)}^{-1}D_{k}^{z}{\left(B_{k}^{R}\right)}^{-1}Z_{k}\right) \end{array}$$

where $B_{k}^{P}$ and $B_{k}^{R}$ are the Cholesky decomposition of $P_{k/k-1}$ and $R_{k}$.

Applying the matrix inversion lemma (${\left(A+BD^{-1}C\right)}^{-1}=A^{-1}-A^{-1}B{\left(D+CA^{-1}B\right)}^{-1}CA^{-1}$), (35) can be become

(36) $${\hat{X}}_{k}={\hat{X}}_{k/k-1}+{\bar{K}}_{k}\left[Z_{k}-H_{k}{\hat{X}}_{k/k-1}\right]$$

where

(37) $${\bar{K}}_{k}={\bar{P}}_{k/k-1}H_{k}^{T}{\left(H_{k}{\bar{P}}_{k/k-1}H_{k}^{T}+{\bar{R}}_{k}\right)}^{-1}$$

where ${\bar{P}}_{k/k-1}=B_{k}^{P}{\left(D_{k}^{x}\right)}^{-1}B_{k}^{P,T}$, ${\bar{R}}_{k}=B_{k}^{R}{\left(D_{k}^{z}\right)}^{-1}B_{k}^{R,T}$. The specific derivation from (35)–(37) can be found in [36].

To estimate the system state through (36), it is necessary to compute the filter gain ${\tilde{K}}_{k}$ in (35) firstly. The calculation of the gain inherently relies on the posterior state estimate ${\hat{X}}_{k}$, which is involved in (33) and (34). This clearly indicates an iterative process for state estimation. Therefore, we outline the measurement update process of the maximum correntropy filter based on the Cauchy kernel as follows:

(i) Let the iteration index *t* = 1 and ${\hat{X}}_{k}^{1}={\hat{X}}_{k/k-1}$, ${\hat{X}}_{k}^{t+1}$ can be achieved by the following process

(38) $${\hat{X}}_{k}^{t+1}={\hat{X}}_{k}^{t}+{\tilde{K}}_{k}\left[Z_{k}-H_{k}{\hat{X}}_{k}^{t}\right]$$

(39) $${\tilde{K}}_{k}={\tilde{P}}_{k/k-1}H_{k}^{T}{\left(H_{k}{\tilde{P}}_{k/k-1}H_{k}^{T}+{\tilde{R}}_{k}\right)}^{-1}$$

(40) $${\tilde{P}}_{k/k-1}=B_{k}^{P}{\left({\tilde{D}}_{k}^{x}\right)}^{-1}B_{k}^{P,T}$$

(41) $${\tilde{R}}_{k}=B_{k}^{R}{\left({\tilde{D}}_{k}^{z}\right)}^{-1}B_{k}^{R,T}$$

where ${\tilde{D}}_{k}^{x}=diag\left(\begin{array}{c} G_{\sigma }({\tilde{E}}_{k}^{1}{)}^{2},\ldots ,G_{\sigma }({\tilde{E}}_{k}^{n}{)}^{2} \end{array}\right)$ and ${\tilde{D}}_{k}^{z}=diag\left(\begin{array}{c} G_{\sigma }({\tilde{E}}_{k}^{n+1}{)}^{2},\ldots ,G_{\sigma }({\tilde{E}}_{k}^{n+m}{)}^{2} \end{array}\right)$; ${\tilde{E}}_{k}^{i}$ is the element of ${\tilde{E}}_{k}$ and ${\tilde{E}}_{k}=Y_{k}-M_{k}{\hat{X}}_{k}^{t}$.

(ii) Compare the posterior estimation from the current iteration ${\hat{X}}_{k}^{t+1}$ with those from the previous iteration ${\hat{X}}_{k}^{t}$. If it satisfies the following condition, the iterative process is terminated; otherwise, set the final state estimation ${\hat{X}}_{k}={\hat{X}}_{k}^{t+1}$ and return to Step (i).

(42) $$\frac{\left\|{\hat{X}}_{k}^{t+1}-{\hat{X}}_{k}^{t}\right\|}{\left\|{\hat{X}}_{k}^{t}\right\|}\leq \varepsilon$$

where ε is a relatively small number.

(iii) Compute the posterior error covariance matrix as

(43) $$P_{k}=(I-{\tilde{K}}_{k}H_{k})P_{k/k-1}$$

At this time, the measurement update process at time *k* of the maximum correntropy filter based on the Cauchy kernel can be terminated.

It can be seen from (33) to (37) that as the bandwidth σ→∞, $D_{k}^{x}$ and $D_{k}^{z}$ will tend to unity matrices. In this case, the maximum correntropy filter based on the Cauchy kernel will be degraded to the Kalman filter and its robustness will gradually vanish. If σ→0, $D_{k}^{x}$ and $D_{k}^{z}$ will become **0**, the calculation of filter gain ${\tilde{K}}_{k}$ will be out of operation and the filter will be disabled. Thus, an appropriate kernel bandwidth is vital to affect the performance of the maximum correntropy filter based on the Cauchy kernel. Currently, it is usually determined by prior information or a multiple test computation prior to the filtering solution.

### 3.2. Event-Driven Conditions

From the above filtering algorithm process, it can be seen that multiple iterations are involved. This means it needs a large computational cost for the filtering calculation at each time. Thus, in the times without non-Gaussian measurement noise, the maximum correntropy filter based on the Cauchy kernel will involve too many redundant calculations. Due to this reason, this section establishes an adaptive event-driven mechanism to effectively recognize the non-Gaussian measurement noise, which can significantly improve the computational efficiency of the maximum correntropy filter based on the Cauchy kernel.

The constructed event-driven conditions are related to the filter’s innovation vector information, which is calculated as [37]

(44) $${\tilde{Z}}_{k}=Z_{k}-H_{k}{\hat{X}}_{k/k-1}$$

In the normal case, the innovation ${\tilde{Z}}_{k}$ should adhere to a Gaussian distribution and its covariance is [37]

(45) $$P_{ZZ,k/k-1}=H_{k}P_{k/k-1}H_{k}^{T}+R_{k}$$

Consider that $P_{ZZ,k/k-1}$ is a positive matrix, we can construct a unitary matrix $L_{k}$ to make the following relationship hold

(46) $$L_{k}^{T}P_{ZZ,k/k-1}L_{k}=\Sigma_{k}$$

where $\Sigma_{k}=diag\left\{\begin{array}{c} \lambda_{k1},\;\lambda_{k2},\ldots ,\lambda_{kn_{i}} \end{array}\right\}\in n_{i}\times n_{i}$, $\begin{array}{c} \lambda_{k1},\;\lambda_{k2},\ldots ,\lambda_{kn_{i}} \end{array}$ are the eigenvalues of $P_{ZZ,k/k-1}$ and $n_{i}$ represents the number of eigenvalues.

Further, define $F_{k}=L_{k}\Sigma_{k}^{-1/2}$, the innovation vector can be normalized as

(47) $${\bar{Z}}_{k}=F_{k}^{T}{\tilde{Z}}_{k}$$

Equation (47) is called Mahalanobis transformation. The elements of ${\bar{Z}}_{k}$ should be uncorrelated and obey the standard Gaussian distribution [22,37]. Thus, the event-driven conditions for the detection of the non-Gaussian measurement noise can be constructed as

(48) $$\tau_{k}=\left\{\begin{array}{l} 1\;\;\;\kappa_{\beta }\leq {\left\|{\bar{Z}}_{k}\right\|}_{\infty }\leq \kappa_{\alpha }\\ 0\;\;\;Otherwise \end{array}\right.$$

where ${\left\|{\bar{Z}}_{k}\right\|}_{\infty }$ represents the infinity norm of ${\bar{Z}}_{k}$, i.e., ${\left\|{\bar{Z}}_{k}\right\|}_{\infty }=\max\left\{{\bar{Z}}_{k,1},\;{\bar{Z}}_{k,2},\ldots ,{\bar{Z}}_{k,q}\right\}$; $\kappa_{\beta }$ and $\kappa_{\alpha }$ are the lower threshold and the higher threshold, respectively.

Based on (48), there are three cases that will appear:

Case 1: ${\left\|{\bar{Z}}_{k}\right\|}_{\infty }<\kappa_{\beta }$ and $\tau_{k}=0$. This case considers that the integration of the gyros/star sensor can be predictable and the measurement does not need to be applied to update the estimation. The purpose of this case is to reduce the communication burden between the gyros and star sensor so as to decrease the whole system’s computational cost.

Case 2: $\kappa_{\beta }\leq {\left\|{\bar{Z}}_{k}\right\|}_{\infty }\leq \kappa_{\alpha }$ and $\tau_{k}=1$. This case considers that the measurement is normal and the non-Gaussian noise is not involved;

Case 3: ${\left\|{\bar{Z}}_{k}\right\|}_{\infty }>\kappa_{\alpha }$ and $\tau_{k}=0$. This case considers that the measurement is deviant and the non-Gaussian noise is involved.

It should be noted that the selection of thresholds $\kappa_{\beta }$ and $\kappa_{\alpha }$ is very important for the detection of the non-Gaussian measurement noise. $\kappa_{\beta }$ is chosen to balance the communication burden and estimation performance, which can be set according to the required communication rate. $\kappa_{\alpha }$ is always set according to the confidence level. Through the simulation test, a 97.5% confidence level can obtain a relatively satisfactory detection performance.

### 3.3. Algorithm Description

Based on the maximum correntropy Kalman filter based on the Cauchy kernel described in Section 3.1 and event-driven conditions constructed in Section 3.2, we can establish the algorithm of the event-driven maximum correntropy Kalman filter based on the Cauchy kernel, whose computational process is illustrated in Algorithm 1. It can balance the computational efficiency and robustness of the filter, reducing unnecessary computational load while ensuring the algorithm’s anti-interference performance.
**Algorithm 1.** Event-driven- maximum correntropy filter based on Cauchy kernel (ED-MCFCK)1**Initialization:** ${\hat{x}}_{0}$, $P_{0}$2**For** *k* = 1,2,…
{3  Compute ${\hat{X}}_{k/k-1}$ and $P_{k/k-1}$ as (22) and (23) of Kalman filtering procedures;4  Calculate filter’s innovation vector as well as its covariance as (44) and (45), and further construct the normalized innovation vector as (47).5  Compute and conduct the event driven condition by (48).6  **If** ${\left\|{\bar{Z}}_{k}\right\|}_{\infty }<\kappa_{\beta }$ **and** $\tau_{k}=0$, set the state prediction ${\hat{X}}_{k/k-1}$ and $P_{k/k-1}$ as the final state estimation.7  **If** $\kappa_{\beta }\leq {\left\|{\bar{Z}}_{k}\right\|}_{\infty }\leq \kappa_{\alpha }$ and $\tau_{k}=1$, execute (24)–(26) to obtain system state esti-mation ${\hat{X}}_{k}$ and $P_{k}$.8  **If** ${\left\|{\bar{Z}}_{k}\right\|}_{\infty }>\kappa_{\alpha }$ **and** $\tau_{k}=0$, (indicating the presence of non-Gaussian noise in the measurement information), the MCFCK is driven.
  {9   Let the iteration index *t* = 1 and ${\hat{X}}_{k}^{1}={\hat{X}}_{k/k-1}$;10    Compute ${\tilde{P}}_{k/k-1}$ and ${\tilde{R}}_{k}$ according to (40) and (41), and further calcu late filter gain ${\tilde{K}}_{k}$ by (39).11    Compute ${\hat{X}}_{k}^{t}$ by (38) and test the *Condition* (42);12    **If** *Condition* ≥ε13    Let *t* = *t* + 1 (iterations) and ${\hat{X}}_{k}={\hat{X}}_{k}^{t+1}$, and conduct the next iteration.14    **Else**, the iterative process is terminated and ${\hat{X}}_{k}={\hat{X}}_{k}^{t}$.
  }15  Compute the posterior error covariance matrix by (43).
}

## 4. Simulation Evaluations and Discussions

Simulation evaluations have been conducted to comprehensively validate the efficacy of the proposed event-driven maximum correntropy filter based on the Cauchy kernel (ED-MCFCK) for a two-dimensional turntable’s spatial orientation using the gyros/star sensor integration. A comparison analysis of the proposed ED-MCFCK with the Kalman filter and MCF based on the Gaussian kernel (abbreviated as MCF) as well as MCFCK is also conducted in this section for the handling of non-Gaussian noise involved in the measurements.

**Figure 3:**
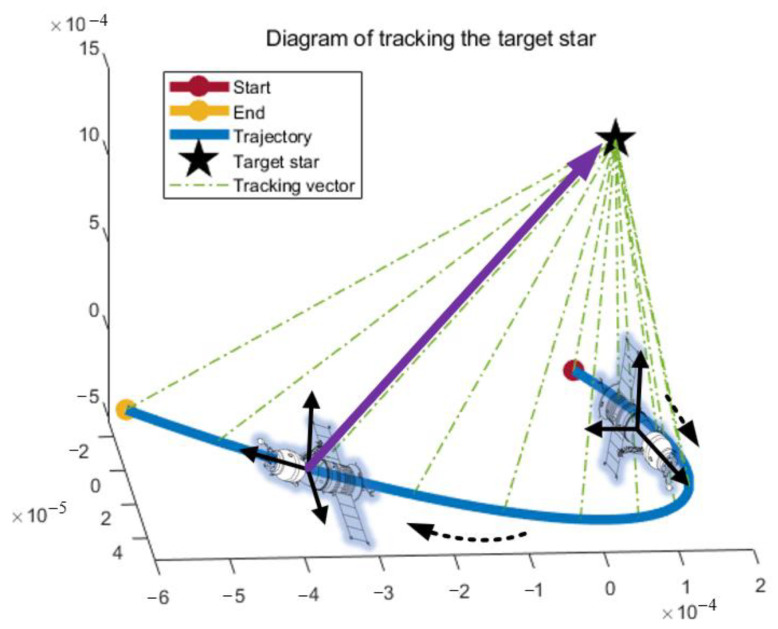
Trajectory and tracking vector of the spacecraft in *I*-frame. The spacecraft’s trajectory is plotted using the endpoint of the tracking vector, so this trajectory is unitless. The black solid arrows represent the spacecraft’s attitude, the green dashed and purple solid arrows represent the tracking vector, and the black dashed arrows represent the spacecraft’s direction of motion.

**Figure 4:**
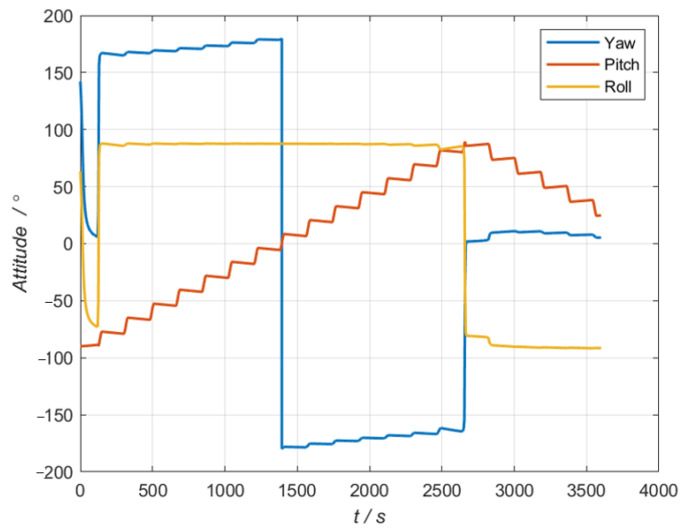
Attitude change of the spacecraft.

As shown in Figure 3, a trajectory of spacecraft is designed according to its actual flight process, which is a classical parabolic curve motion. Further, the attitude of the spacecraft at every time step, including yaw, pitch, and roll is depicted in Figure 4. The initial attitude of the spacecraft was (142.16°, −89.78°, 63.16°) and the total simulation time was 3600 s. Accordingly, a two-dimensional turntable was installed on the spacecraft, and the tracking vector between the spacecraft and the target star is also shown in Figure 3.

For the filtering loop shown in Figure 2, the gyros’ sampling rates are both 20 Hz, the star sensor’s sampling rate is 1 Hz, and the other simulation parameters are listed in Table 1. The initial state and its error covariance, as well as the initial process noise covariance, are set as

(49) $$\left\{\begin{array}{l} X_{0}={\left[0_{6\times 1}\right]}^{T}\\ P_{0}=diag[\begin{array}{ccc} {\left(1rad\right)}^{2} & {\left(1rad\right)}^{2} & \begin{array}{cccc} {\left(1rad\right)}^{2} & {\left(1\prime \prime \right)}^{2} & {\left(1\prime \prime \right)}^{2} & {\left(1\prime \prime \right)}^{2} \end{array} \end{array}]\\ Q_{0}=diag[\begin{array}{ccc} {\left(0.01^{\circ }/\sqrt{h}\right)}^{2} & {\left(0.01^{\circ }/\sqrt{h}\right)}^{2} & {\left(0.01^{\circ }/\sqrt{h}\right)}^{2} \end{array}] \end{array}\right.$$

A. Accuracy Evaluation and Analysis

To verify the accuracy and robustness of the proposed ED-MCFCK in the presence of non-Gaussian measurement noise, two typical non-Gaussian measurement noise scenarios were considered, i.e., non-Gaussian noise with outliers in measurements and α stable noise in measurements.

By using the trial and error method [38], the bandwidth parameters σ in the above-mentioned maximum correntropy filters were determined to be 13. For the designed event-driven conditions, the confidence level is set as 97.5% (α = 0.025) with 3 degrees of freedom (m = 3).

(1) Non-Gaussian noise with outliers in measurements

To simulate this situation, a random noise with Gaussian distribution was directly injected into the measurement during the time interval (1500 s, 2500 s) with a probability of ρ=0.5. Thus, the measurement becomes the following

(50) $$Z_{k}=\left\{\begin{array}{l} Z_{k},\;\;\;\;\;\;\;\;\;\;\;\;\;\;others\\ \left(1-\rho )Z_{k}+\rho [Z_{k}+N(0,{\left(4\times 10^{-4}rad\right)}^{2})],\;(1500s,2500s\right) \end{array}\right.$$

It can be seen that the measurement noise in (50) is mixed Gaussian, which is a non-Gaussian and strong impulsive noise (outliers).

**Figure 5:**
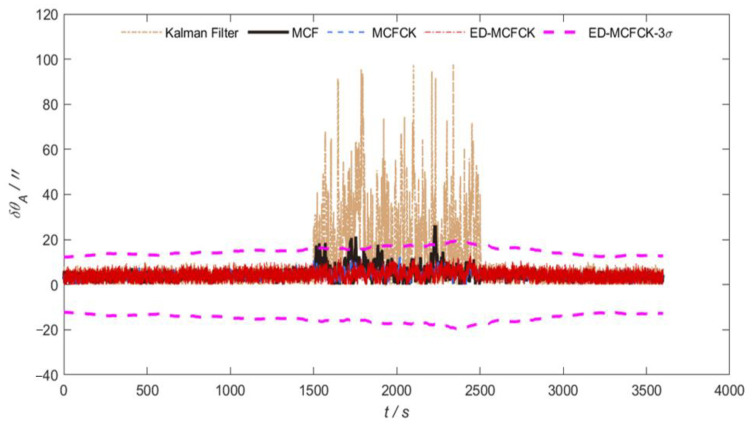
Orientation errors in azimuth and 3σ error boundary for the case of non-Gaussian noise with outliers.

**Figure 6:**
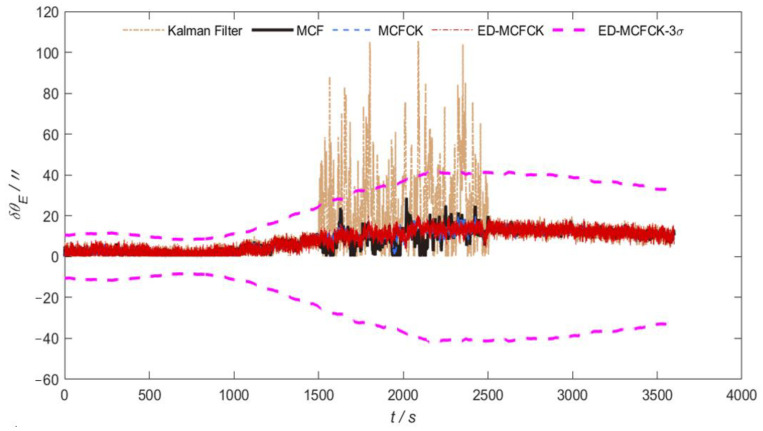
Orientation errors in pitch and 3σ error boundary for the case of non-Gaussian noise with outliers.

For the case of non-Gaussian noise with outliers in measurements, Figure 5 and Figure 6 illustrate the orientation errors in azimuth and pitch achieved by the above-mentioned Kalman filter, MCF, MCFCK and ED-MCFCK, respectively. Further, the corresponding 3σ error boundary for the ED-MCFCK, which is calculated based on the 10 runs of Monte Carlo simulations as in [39], is also provided in Figure 5 and Figure 6. As shown in Figure 5 and Figure 6, during the time interval (1500 s, 2500 s) with non-Gaussian noise, the Kalman filter has no ability and fails to handle the abnormal non-Gaussian noise. This further deteriorates the obtained state estimate during the filtering measurement update process, resulting in a significant degradation in orientation accuracy. The orientation errors in azimuth and pitch exhibit severe fluctuations. In contrast, MCF, MCFCK and ED-MCFCK can leverage the robustness of the Gaussian/Cauchy kernel to effectively handle the non-Gaussian noise information, leading to stable orientation errors in azimuth and pitch.

The global orientation accuracy and the corresponding Root Mean Square Error (RMSE) are further used to evaluate the orientation accuracy, where the global orientation error is defined as

(51) $$\left\|\delta \theta \right\|=\sqrt{\delta \theta_{A}^{2}+\delta \theta_{E}^{2}}$$

where $\delta \theta_{A}$ and $\delta \theta_{E}$ are the azimuth error and pitch error of the turntable, respectively.

The RMSE at time *k* is defined as

(52) $$RMSE=\sqrt{\frac{1}{N}\sum_{i=1}^{N}{\left|\delta \theta_{k}\right|}^{2}}$$

where *N* is the total simulation time, and $\delta \theta_{k}$ represents the orientation error at time *k*.

Table 2 shows the mean RMSEs of global orientation error as well as the azimuth and pitch errors for each filter. Due to the ability of robustness on non-Gaussian noise, the mean RMSEs of global orientation error as well as the azimuth and pitch errors achieved by the MCF are 55.8%, 67.76% and 48.46% lower than those of the Kalman filter. Meanwhile, the mean RMSEs of global orientation error as well as the azimuth and pitch errors achieved by the MCFCK are 56.26%, 70.35% and 47.97% lower than those of the Kalman filter. Further, the mean RMSEs of global orientation error as well as the azimuth and pitch errors achieved by the ED-MCFCK are 56.91%, 70.47% and 48.84% lower than those of the Kalman filter. These indicate that the MCF, MCFCK and ED-MCFCK exhibit a similar estimation performance for the case of non-Gaussian noise with outliers in measurements and they have a better sensitivity to the non-Gaussian measurement noises compared to the Kalman filter.

(2) α stable noise in measurements

The α stable noise is a significant type of non-Gaussian random distribution, and its two most important features are the stability of the probability distribution and the heavy-tailed probability density function. The characteristic function of the α stable noise is given by [40]

(53) $$\varphi (t)=\exp\{j\delta t-\alpha {\left|t\right|}^{\beta }[1+j\zeta \mathrm{sgn}(t)\omega (t,\beta )]\}$$

with

(54) $$\omega (t,\beta )=\left\{\begin{array}{c} \tan(\frac{\beta \pi }{2}),\beta \neq 1\\ \frac{2}{\pi }\log\left|t\right|,\beta =1 \end{array}\right.$$

(55) $$\mathrm{sgn}(t)=\left\{\begin{array}{l} 1,\;\;t\text{>}0\\ 0,\;\;t=0\;\\ -1,\;\;\;t\text{<}0 \end{array}\right.$$

where β∈(0,2] is the characteristic index, ζ∈[−1, 1] is the symmetry parameter, δ is the location factor and α>0 is the dispersion factor.

To test the performance of the four filters under α stable noise, the above noise form was injected into the measurement during the time intervals (1000 s, 1500 s) and (2500 s, 3000 s), where (β, ζ, α, δ)=(1.8, 0, 1.6, 0) is set in this simulation.

**Figure 7:**
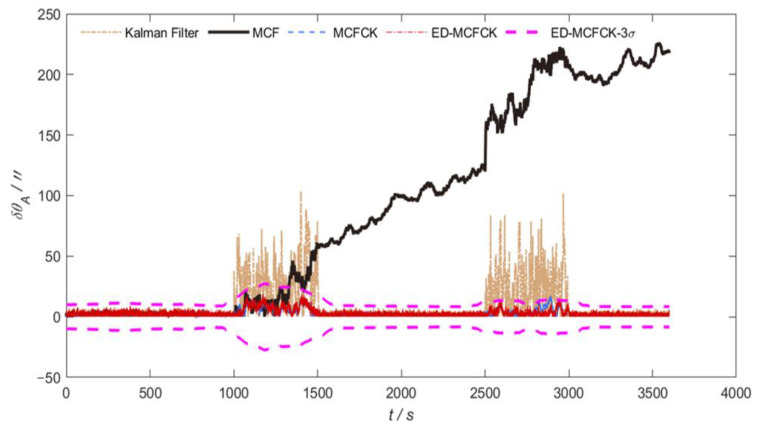
Orientation errors in azimuth and 3σ error boundary for the case of σ stable noise.

**Figure 8:**
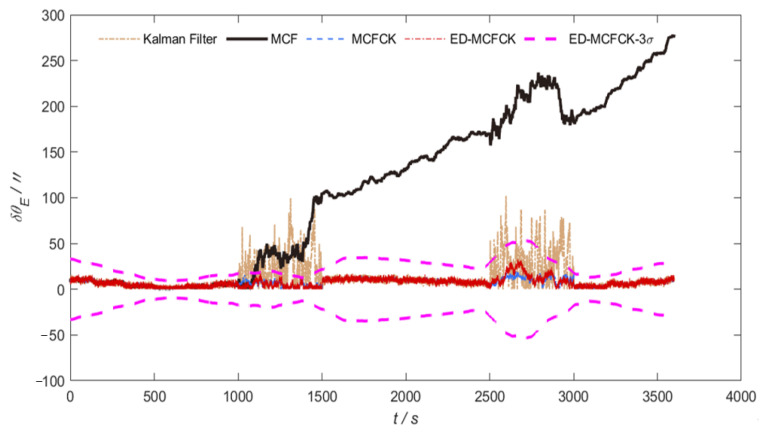
Orientation errors in pitch and 3σ error boundary for the case of σ stable noise.

Figure 7 and Figure 8 provide the orientation errors in azimuth and pitch achieved by the Kalman filter, MCF, MCFCK and ED-MCFCK as well as the 3σ error boundary of ED-MCFCK for the case of α stable noise in measurement. It can be seen that as stated previously, MCF is unable to handle heavy-tailed noise anomalies due to the limitation of the Gaussian kernel function; thus, its estimation error is divergent. In contrast, MCFCK and ED-MCFCK can effectively handle the influence of α stable noise with a heavy-tailed characteristic through the robustness of the Cauchy kernel. They show similar performance with the case of non-Gaussian noise with outliers in measurements and have a stronger robustness than the Kalman filter. Table 3 shows the mean RMSEs of global orientation error as well as the azimuth and pitch errors by the above four filters. Due to the robustness of the Cauchy kernel of MCFCK and ED-MCFCK, the mean RMSEs of the global orientation error as well as the azimuth and pitch errors achieved by the MCFCK are 63.19%, 78.65% and 53.46% lower than those of the Kalman filter, and the mean RMSEs of ED-MCFCK are 66.43%, 81.97% and 56.98% lower than those of the Kalman filter. However, due to the limitation of the Gaussian kernel in handling the heavy-tailed noise anomalies, the mean RMSEs of global orientation error as well as the azimuth and pitch errors by the MCF increased by 2008.05%, 3599.69% and 1625.61% compared the ED-MCFCK. Therefore, it is evident that the proposed ED-MCFCK has a superior robust performance to curb heavy-tailed non-Gaussian noise such as α stable noise, leading to improved orientation accuracy in the complex space environment.

B. Computational Performance Evaluation

**Figure 9:**
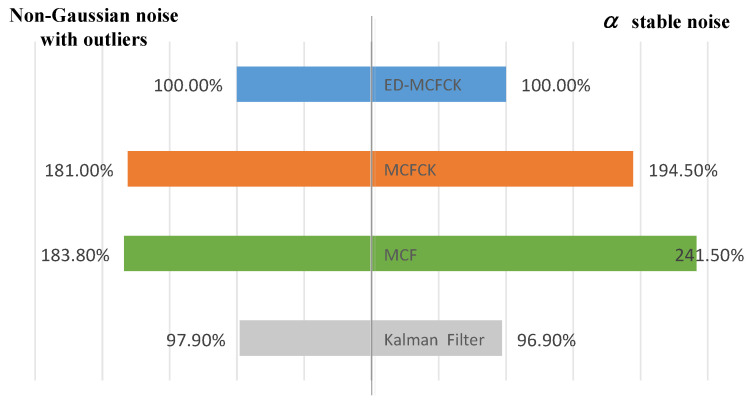
Relative efficiencies of all the filters compared to the ED-MCFCK based on the simulation cases. The blue, orange, green, and gray histograms respectively represent the computational efficiency of the ED-MCFCK, MCFCK, MCF, and Kalman Filter algorithms.

The computational efficiency of the proposed ED-MCFCK is also discussed by comparison with the Kalman filter, MCF and MCFCK via a Monte Carlo simulation. It was conducted 10 times on an Intel(R) Core(TM) i5-9300H 2.40 GHz PC with 8 GB memory to study the computational performance of the proposed ED-MCFCK based on the above two types of non-Gaussian measurement noise scenarios. The computational times for each Monte Carlo run of the Kalman filter, MCF, MCFCK and ED-MCFCK are listed in Table 4. Further, the relative efficiencies of all the filters compared to the ED-MCFCK are also computed and shown in Figure 9.

It can be seen from Table 4 and Figure 9 that the Kalman filter has optimal real-time performance and its computational time for each Monte Carlo run is smallest among all the four filters. This is the reason why it is widely used in engineering practices. Further, the computational times of MCF and MCFCK are significantly longer than those of the Kalman filter. The filtering computational time of MCF is 87.8% (for the first case) and 149.3% (for the second case) longer than that of the Kalman filter. The filtering computational time of MCFCK is 84.9% (for the first case) and 100.7% (for the second case) longer than that of the Kalman filter. They have a similar computational performance since multiple iterations are involved in filtering every time and result in unnecessary redundant calculations. Both MCF and MCFCK’s real-time performance are poor; thus, they are not suitable for the practice applications. As a comparison, the proposed ED-MCFCK establishes a flexible event-driven mechanism to detect and process the case of non-Gaussian measurement noise. This effectively avoids the unnecessary redundant calculations and significantly improves the computational efficiencies of MCF and MCFCK. The ED-MCFCK has an approximated computational efficiency compared to the Kalman filter (97.9% for the first case and 96.9% for the second case). Thus, through the designed event-driven mechanism, ED-MCFCK can simultaneously obtain a good robustness on non-Gaussian measurement noise and a strong real-time performance in practical engineering.

The results of the above simulation evaluations and analysis demonstrate that the proposed ED-MCFCK not only has a superior ability to deal with the influence of non-Gaussian measurement noise but can also achieve superior real-time spatial applications by avoiding unnecessary computational costs in time points without non-Gaussian measurement noise, leading to improved spatial orientation performance for the gyros/star sensor integration used in the two-dimensional turntable structure.

## 5. Conclusions

This paper proposes an event-driven maximum correntropy filter based on the Cauchy kernel to improve the performance of gyros/star sensor integration used for spatial orientation under the environment of non-Gaussian measurement noise. The main contributions of this paper can be concluded as follows: (i) it establishes and derives a direct installation mode of gyros/star sensor integration as well as its mathematical model to ensure the turntable control stability; (ii) it further proposes an event-driven maximum correntropy filter based on the Cauchy kernel to handle the influence of non-Gaussian measurement noise on the above gyros/star sensor integration, in which an event-driven mechanism is also constructed based on the innovation information to reduce the unnecessary computational costs involved in the filtering solution. The results of the simulations and comparison analysis indicate that the proposed methodology can effectively curb the non-Gaussian measurement noise through the maximum correntropy criterion based on the Cauchy kernel, and it also can avoid the unnecessary computational cost of MC filters such as MCF and MCFCK, leading to improved robustness for the gyros/star sensor integration of spatial orientation in comparison with the conventional Kalman filter and MC filters.

Future research will focus on the combination of the proposed ED-MCFCK with advanced intelligent methods such as neural networks or deep learning to improve its robustness by adaptively adjusting the kernel bandwidth in real-time to accommodate the complex spatial environment. Further, it will also concentrate on the practical/extension application of the ED-MCFCK in the fields of vehicle navigation or target tracking.

## Figures and Tables

**Table 1:** Simulation parameters of gyros/star sensor integration.

Information Sources	Simulation Parameters	Values
Gyros	Gyros’ constant drift	0.1°/h
	Gyros’ random walk coefficient	0.01°/$\sqrt{h}$
	Scale factor error	100 PPM
	Misalignment error	5″
Star sensor	Goniometric error	5″ (3σ)

**Table 2:** Mean RMSEs of the orientation errors by Kalman filter, MCF, MCFCK and ED-MCFCK for the case of non-Gaussian noise with outliers.

Filtering Methods	Global Orientation Error (″)	Azimuth Error (″)	Pitch Error (″)
Kalman Filter	24.60	16.22	18.49
MCF	10.87	5.23	9.53
MCFCK	10.76	4.81	9.62
ED-MCFCK	10.6	4.79	9.46

**Table 3:** Mean RMSEs of the orientation errors by Kalman filter, MCF, MCFCK and ED-MCFCK for the case of σ stable noise.

Filtering Methods	Global Orientation Error (″)	Azimuth Error (″)	Pitch Error (″)
Kalman Filter	26.27	18.08	19.06
MCF	185.93	120.61	141.5
MCFCK	9.67	3.86	8.87
ED-MCFCK	8.82	3.26	8.20

**Table 4:** The computational times for each Monte Carlo run of the Kalman Filter, MCF, MCFCK and ED-MCFCK based on the simulation cases.

Filtering Methods	Non-Gaussian Noise with Outliers	α Stable Noise
	Computational Time	Computational Time
Kalman Filter	2.78 s	2.80 s
MCF	5.22 s	6.98 s
MCFCK	5.14 s	5.62 s
ED-MCFCK	2.84 s	2.89 s

## Data Availability

The original contributions presented in the study are included in the article; further inquiries can be directed to the corresponding author.

## Abbreviations

**Abbreviations**	**Full Names**
PF	Particle filter
GSF	Gaussian sum filter
MCC	maximum correntropy criterion
MC	maximum correntropy
ED-MCFCK	event-driven maximum correntropy filter based on Cauchy kernel
MCF	maximum correntropy filter
MCFCK	maximum correntropy filter based on Cauchy kernel
RMSE	Root Mean Square Error
